# Radical Molecular Modulator for High-Performance Perovskite Solar Cells

**DOI:** 10.3389/fchem.2020.00825

**Published:** 2020-09-11

**Authors:** Qi Peng, Xin Zheng, Xiaoru Zhang, Shuai You, Lin Li, Yang Zhao, Shujing Zhang, Long Luo, Haipeng Zeng, Xiong Li

**Affiliations:** Michael Grätzel Center for Mesoscopic Solar Cells, Wuhan National Laboratory for Optoelectronics, Huazhong University of Science and Technology, Wuhan, China

**Keywords:** perovskite solar cell, passivation, radical, Lewis adduct, TEMPO

## Abstract

The long-term stability of perovskite solar cells (PSCs) remains an issue impeding their commercialization. Generally, polycrystalline perovskite thin films have many defects on the grain boundaries, which affect the optoelectronic performance and stability of the devices under moisture, heat, illumination, and the presence of an electric field condition. The O-donor Lewis base is often employed to regulate the performance of PSCs such as carbonyl and carboxyl compounds. Herein, we have developed a concept of radical molecular modulation using the O-donor group for high-performance perovskite photovoltaic devices. The judiciously designed radical modulators 2,2,6,6-tetramethyl-1-piperidinyloxy (TEMPO), which located at the perovskite grain boundary through interaction with the perovskite surface sites, effectively passivated the surface defects while templating the formation of large grain crystal and high-quality perovskite thin films. Accordingly, the optimized TEMPO-modulated PSCs achieved a power conversion efficiency of 20.73% with superior stability. This work makes an important contribution for exploring the effect of radical in perovskites to improve the performance of PSCs and other optoelectronic devices.

## Introduction

Organic–inorganic metal halide perovskite material has attracted tremendous attention as a promising renewable energy photovoltaic material due to its excellent properties of high absorption coefficient, tunable direct band gap, low exciton binding energy, long charge carrier lifetime, and low recombination rates (Etgar et al., 2012; Kojima et al., 2012; Lee et al., 2012; Stranks et al., 2013; Xing et al., 2013; Yin et al., 2014; Dong et al., 2015). Interface engineering (Deng et al., 2018; Kim et al., 2018; Jin et al., 2020), crystal engineering (Wu et al., 2020), compositional engineering (Hong et al., 2017; Turren-Cruz et al., 2018; Zhao et al., 2019), and passivation techniques (Tan et al., 2017; Chen et al., 2019) have been developed to improve performance of the perovskite solar cells (PSCs). The bulk and surface trap state of the perovskite active layer is reduced, resulting in a record power conversion efficiency (PCE) of up to 25.2%.

Grain boundaries consist of structural defects, such as undercoordinated Pb^2+^, which cause a non-radiative recombination process and decrease the *V*_OC_ of PSCs (Chen et al., 2011; Zhang et al., 2016, 2019; Sherkar et al., 2017). The passivation of defects and interface traps in perovskite films by functional additives has been proven to be effective approaches to reduce non-radiative recombination and to improve PSC performance (Lin et al., 2017; Zhang et al., 2017; Ma et al., 2019; Zhang and Zhu, 2020). Among them, Lewis bases can bind to the positively charged undercoordinated Pb^2+^ ions, passivating the undercoordinated defect such as pyridine (N-donor), thiophene (S-donor), and carboxyl (O-donor) derivatives (Noel et al., 2014; Lee et al., 2017; Liu et al., 2018; Yang et al., 2018; Zhang et al., 2018). In principle, these molecules have lone pair electrons on their HOMO, while lead ions have “empty orbitals” that accept lone pair electrons forming a Lewis adduct through the coordinate covalent bonds.

**Figure 1:**
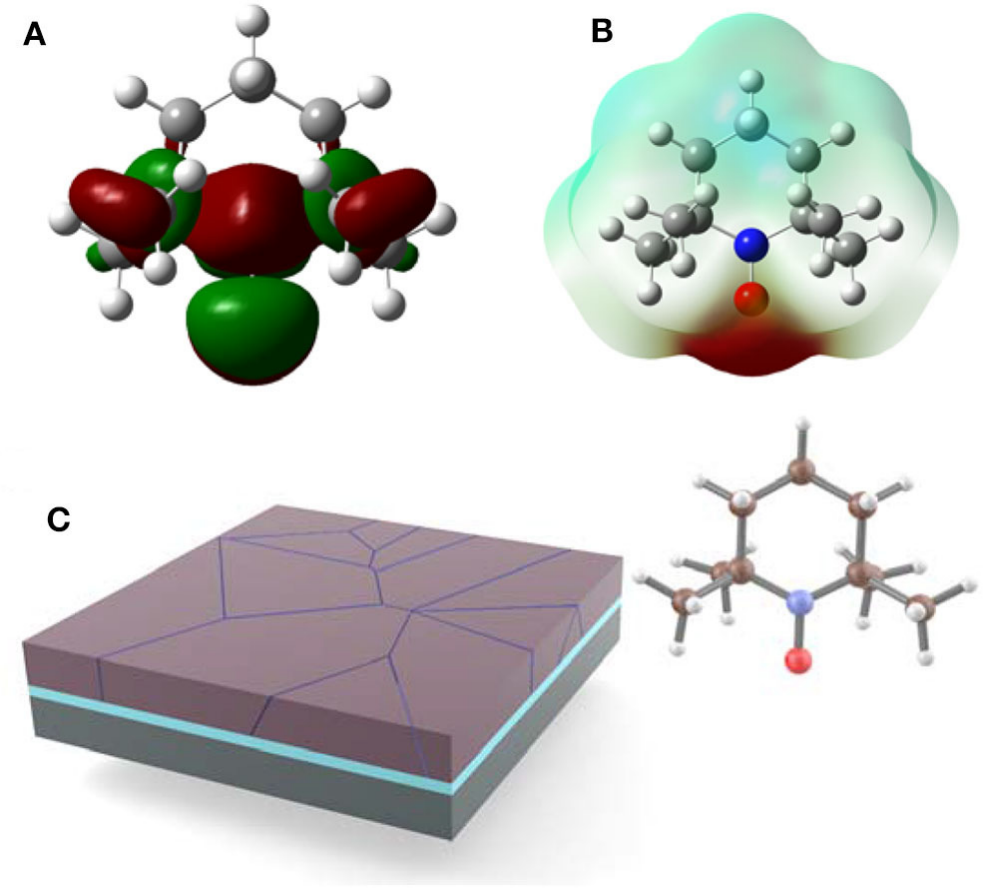
**(A)** Schematic diagram of molecular SOMO distribution diagram of TEMPO. **(B)** Calculated electrostatic potential profiles of TEMPO. **(C)** Schematic illustration of perovskite film with TEMPO.

Aside from the above “normal” molecules, there's another special molecule that contains singly occupied molecular orbitals (SOMO) which are highly reactive intermediates that react with most organic molecules, so-called radical compounds (Tebben and Studer, 2011). Nevertheless, nitroxyl radicals such as 2,2,6,6-tetramethyl-1-piperidinyloxy (TEMPO) are stable due to the steric hindrance effect of adjacent groups (Studer and Curran, 2016). As shown in Figure 1, the electron-deficient region is mainly distributed in the carbon ring, while the nitroxide groups in the electron-rich region have the ability to coordinate with metal ions (Luneau, 2020). Thus, TEMPO exerts a Lewis basic character by acting as a ligand for the undercoordinated Pb^2+^ cations in perovskite.

Recently, Gong employed a chemically robust and electronically active radical polymer to modify the poly(3,4-ethylenedioxythiophene): poly(sodium-*p*-styrenesulfonate) (PEDOT:PSS) and achieved high performance (Zheng et al., 2017). Li's study shows that dopamine-doped PEDOT:PSS has a higher free radical content and a higher charge extraction capability (Huang et al., 2017). However, the passivation mechanism of perovskite by radical has not yet been established. In this paper, we introduce a small radical molecule TEMPO into the PSCs. The addition of TEMPO significantly reduces the defect state density of perovskite thin films, improving the PCE from 19.22 to 20.73%. Our results show that the addition of a small amount of free radicals can effectively improve the performance of PSCs, which provides a new strategy for the regulation of perovskite.

## Result and Discussion

To investigate the effect of radical molecules on the performance of PSCs, we added different concentrations of TEMPO into the perovskite precursor solutions to optimize the doping amount on their properties. The ultraviolet–visible (UV–vis) absorption was employed to study the optical characterization of perovskite films. As shown in Figure S1, all films have a similar absorption intensity and onset value on spectra, which means that TEMPO has almost no effect on the band gap of the perovskites. X-ray diffraction (XRD) patterns of the above films are shown in Figure S2. It was observed that the peaks of perovskite films are mainly located at 13.98° and 28.17°, which can be ascribed to the (001) and (002) crystal planes of perovskite, respectively. The increased intensity of the diffraction peaks at 13.98° and 28.17° of the perovskite film modified with TEMPO indicated that the perovskite crystallinity was improved. We then performed the surface morphology of perovskite films modified with and without TEMPO by scanning electron microscopy (SEM) measurements (Figure S3). It was observed that the grain size and crystal quality of perovskite films with TEMPO were improved with a featureless morphology. The results of SEM and XRD confirm that the addition of TEMPO had an impact to a certain extent in assisting film growth. However, these subtle effects are not the key factor for the significant improvement in *V*_OC_.

**Figure 2:**
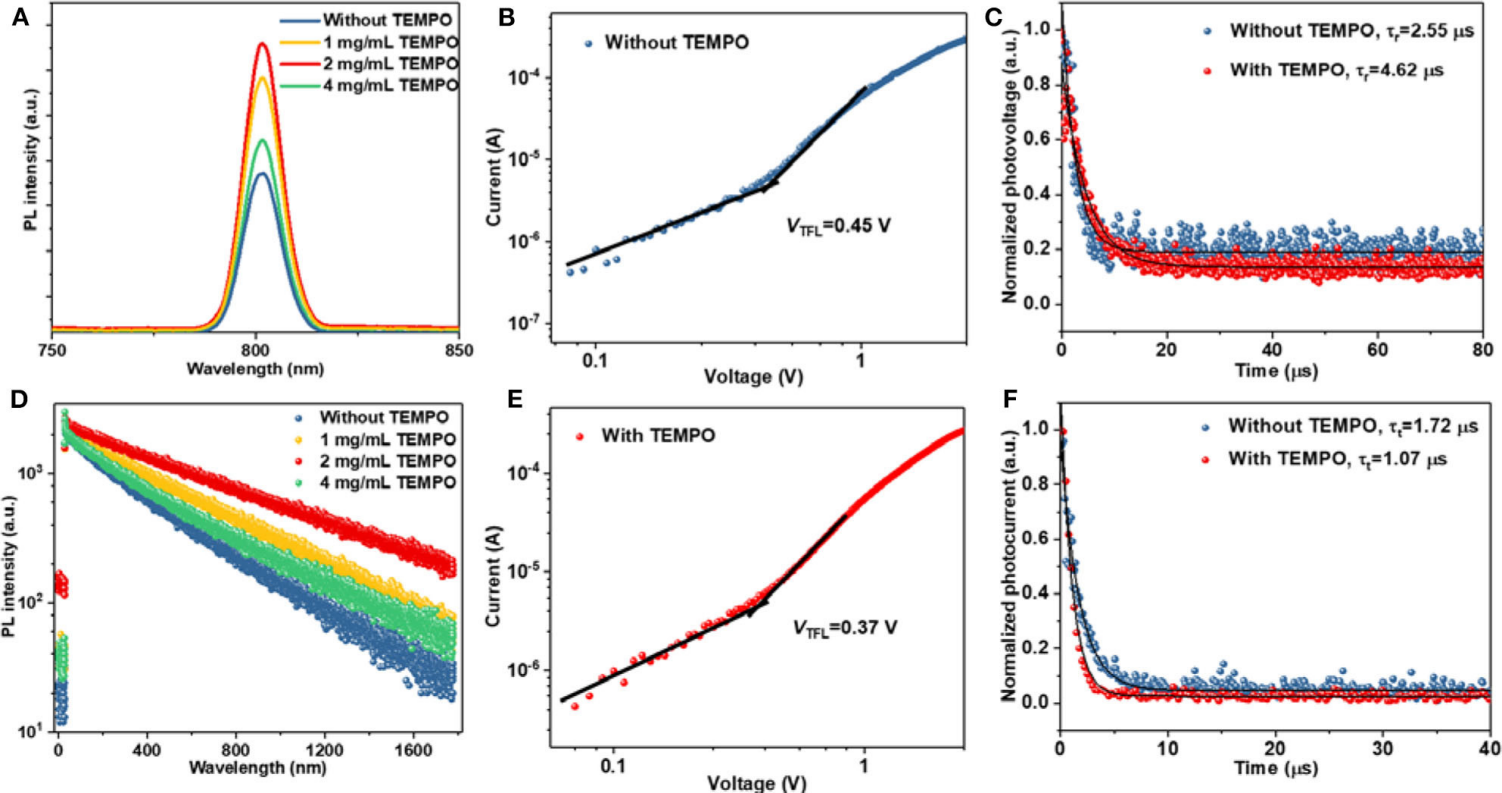
**(A)** Steady-state PL spectra of the perovskite films modified without and with TEMPO. **(B)** The *J*–*V* curves of the hole-only devices based on the structure of ITO/PEDOT:PSS/perovskite/PTAA/Au. **(C)** Transient photovoltage decay curves of the perovskite films modified without and with TEMPO. **(D)** TRPL spectra of the perovskite films modified without and with TEMPO. **(E)** The *J*–*V* curves of the hole-only devices based on the structure of ITO/PEDOT:PSS/perovskite (modified with TEMPO)/PTAA/Au. **(F)** Transient photocurrent decay curves of the perovskite films modified without and with TEMPO.

The Lewis acid–base chemistry technique can effectively improve the performance and stability of halide perovskite through passivating defects. Thus, we investigate the trap density on the photophysical properties of perovskite films. As shown in Figures 2A,D, steady-state photoluminescence (PL) and time-resolved PL (TRPL) were employed to evaluate the dynamics of charge recombination, and the result was summarized in Table S1 in the Supplementary Material. The corresponding perovskite films were deposited on the bare glass substrate. Compared with those of the pristine perovskite film, the intensities of the PL peaks gradually increased with the addition of TEMPO, indicating a reduction of non-radiative recombination in the perovskite films. The TRPL curves (Figure 2D) were fitted to a biexponential decay equation of *I*(*t*) = *I*0 + A1exp(–*t*/τ1) + A2exp(–*t*/τ2), where τ1 and τ2 are the fast and slow decay lifetimes and A1 and A2 are the relative amplitudes, respectively. The fast decay process originates from the Shockley–Read–Hall (SRH) recombination induced by charge-trapping defect states. The proportion of fast decay lifetimes for the modified perovskite decreased from 4.25 to 1.97%. The decay lifetimes are 159.57 and 705.18 ns, respectively, obtaining an average value of 702.71 ns, while the pristine perovskite film lifetimes are 82.19 and 454.13 ns with an average lifetime of 451.17 ns. The higher steady-state PL and the longer PL lifetime indicate that the density of trapped states is lower. This result suggested that TEMPO reduced the density of non-radiative recombination and traps in perovskite films.

To quantitatively analyze the defect density, the perovskite films modified with and without TEMPO have been measured based on a space-charge limited current (SCLC) model. Figures 2B,E show the dark *J*–*V* curves of the hole-only structure of ITO/PEDOT:PSS/perovskite (without and with TEMPO)/PTAA/Au which included an ohmic regime (*n* = 1), a trap-filled limited regime (TFL, *n* > 3), and a space-charge limited current (SCLC, *n* = 2) regime (child). The trap states density was determined by the trap-filled limit voltage (*V*_TFL_) using the following equation:

$$\begin{array}{l} N_{\text{t}}=\frac{2\varepsilon \varepsilon_{\text{0}}}{eL^{2}}V_{\text{TFL}} \end{array}$$

where ε is the dielectric constant, ε_0_ is the vacuum permittivity, *e* is the charge constant, and *L* is the thickness of the perovskite film. The hole trap density in the TEMPO-modified perovskite is calculated to be 3.38 × 10^15^ cm^−3^, which is much lower than that of control devices (4.11 × 10^15^ cm^−3^). The significant decrease on trap density illustrates that the hybrid perovskite layer can be effectively passivated by TEMPO.

To further explore the reason for the improvement of perovskite properties by TEMPO, charge-recombination lifetime (τ_r_) and charge-transport time (τ_t_) were obtained from the measurements of the corresponding transient photovoltage (TPV) and photocurrent decay (TPC), respectively (Figures 2C,F) (Tress et al., 2013). The τ_r_ significantly increased from 2.55 μs for the control device to 4.62 μs for the TEMPO-modified device. The increment of τ_r_ could be attributed to the slower recombination of surface charges in TEMPO-modified devices, which was consistent with the TRPL measurement. In addition, the transient signal of TPC, measured at short-circuit condition, offered novel insights into the carrier extraction and charge trapping in the device. As shown in Figure 2F, the τ_t_ of the TEMPO-modified device (1.07 μs) was shorter than that of the control device (1.72 μs), suggesting that photogenerated charge carriers in perovskite layers can be extracted and transferred to the hole (electron) transport layer in time. This result indicated that radical molecules could reduce the defects in the TEMPO-modified PSCs. Thus, the device with TEMPO exhibits a higher V_OC_.

**Figure 3:**
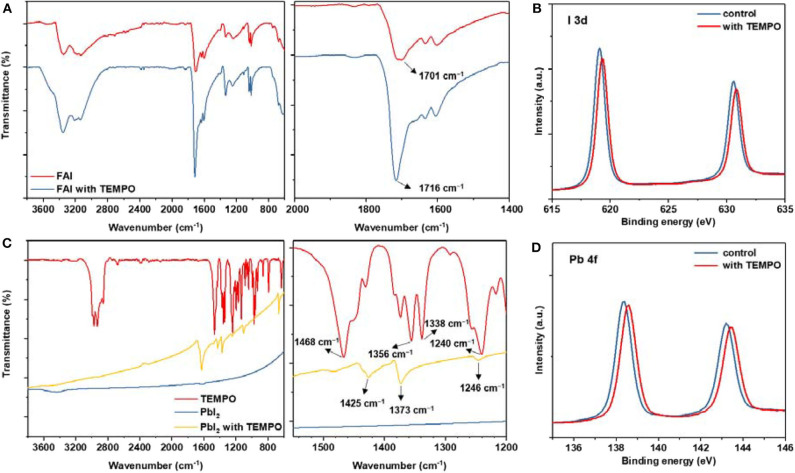
**(A)** FTIR spectra of pure FAI and FAI-TEMPO. **(B)** FTIR spectra of pure TEMPO, PbI_2_, and PbI_2_-TEMPO. **(C)** XPS spectra of I 3d of perovskite layers with and without TEMPO. **(D)** XPS spectra (Pb 4f) of perovskite films with and without TEMPO.

To figure out the mechanism behind these improvements, we comparatively show the FTIR spectra of neat FAI and FAI/TEMPO (in powder). Overall, the signals at 1,701 cm^−1^ were assigned to the stretch vibration of C=N. As shown in Figure 3A, after TEMPO was added, the peak of C=N belonging to FAI shifts to a higher wavelength. This spectroscopic feature points to the hydrogen bond interaction between FAI and the N–O radical in TEMPO. Furthermore, the FTIR spectra of neat TEMPO, PbI_2_, and PbI_2_/TEMPO (in powder) were shown in Figure 3B for comparison. As shown in the magnified spectra in Figure 3B, the signals of nitroxyl radical have an obvious shift, which indicates the coordination interaction between TEMPO and Pb^2+^. Then, we further investigated the chemical states of Pb and I by X-ray photoelectron spectroscopy (XPS). As compared with that of a pristine perovskite film, the binding energy (BE) of Pb 4f peaks and I 3d peaks of the perovskite films with TEMPO are gradually increased by 0.2 eV. Slight BE shifts for Pb 4f indicate that TEMPO directly interacts with Pb (Figures 3C,D).

**Figure 4:**
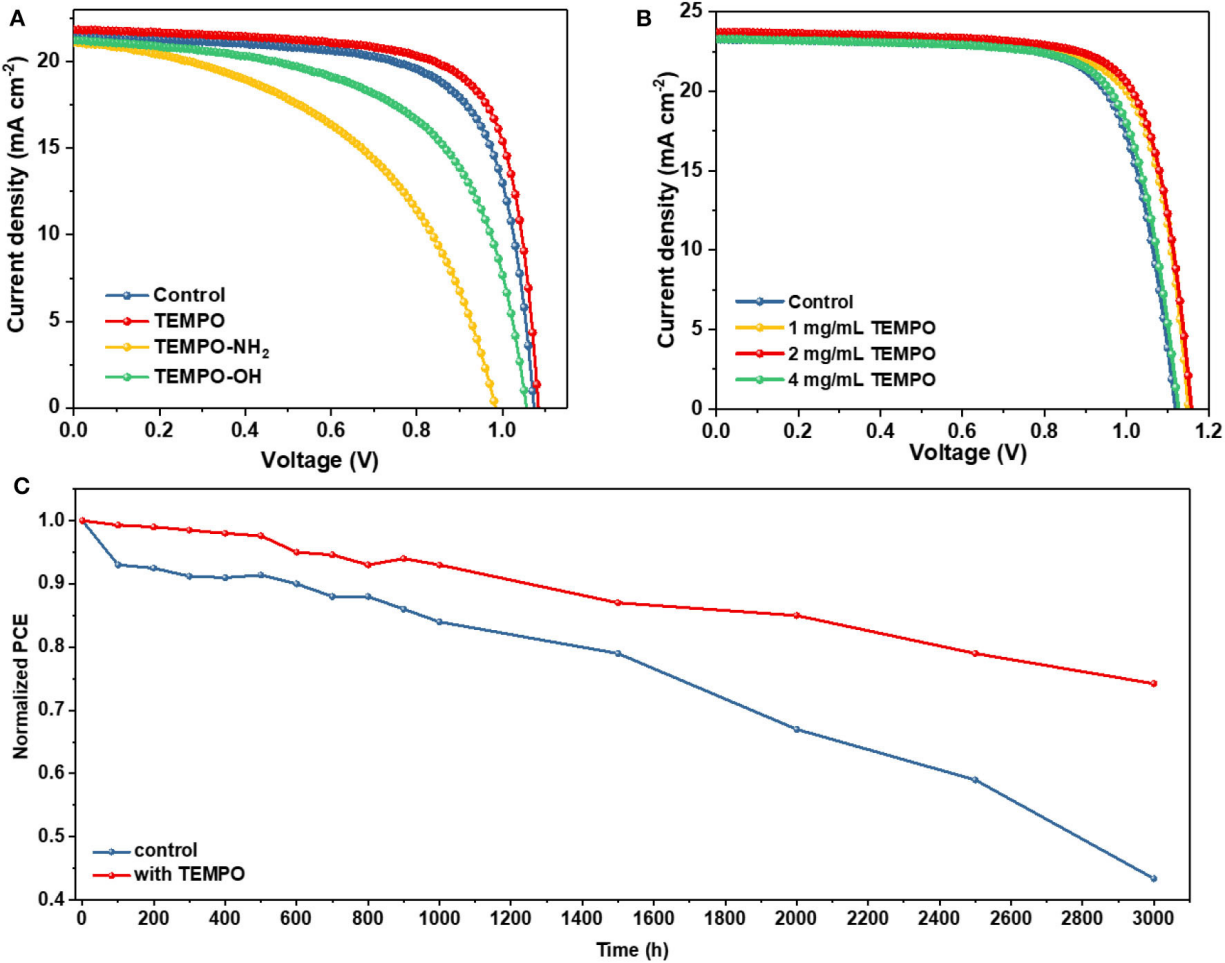
**(A)** *J*–*V* curves of the devices based on control and different molecules passivating perovskite films. **(B)** *J*–*V* curves of the champion devices with different TEMPO molar ratio under 1 sun illumination. **(C)** The environmental stability of the unencapsulated devices under 50% humidity at room temperature.

We explored the effects of radical small molecules with different functional groups on the optoelectronic properties of PSCs. The current density–voltage (*J*–*V*) curves (reverse scan) for the control device and the devices modified with different molecules are shown in Figure 4A, and the corresponding photovoltaic parameters are listed in Table S2. The concentration of three radical small molecules with different functional groups is 1 mg ml^−1^. The control device exhibited a PCE of 16.18% with 21.45 mA cm^−2^ of short-circuit current density (*J*_SC_), 1.073 V of open-circuit voltage (*V*_OC_), and 70.3% of fill factor (FF). Unfortunately, the modification by TEMPO-NH_2_ and TEMPO-OH had a negative effect on the photoelectric performance of the device with significantly decreased *V*_OC_ and FF. In contrast, the device modified with TEMPO exhibited a much higher PCE of 17.33% with a *J*_SC_ of 21.87 mA cm^−2^, *V*_OC_ of 1.084 V, and FF of 73.1%.

Then we optimized the modification concentration of TEMPO. Figure 4B displays the *J*–*V* curves (reverse scan) of PSCs modified with different concentrations of TEMPO. Detailed parameters of different kinds of PSCs are summarized in Table 1. The *V*_OC_, *J*_SC_, and FF all become enlarged with the modification of TEMPO. The control device shows a PCE of 19.22%, with a *J*_SC_ of 23.26 mA cm^−2^, *V*_OC_ of 1.119 V, and FF of 73.8%. The performance metrics exhibit an increase when incrementally varying the TEMPO modification concentrations. From Figure 4B, we establish an optimal concentration of 2 mg ml^−1^ for the TEMPO modification concentration. In this condition, the best PCE of PSCs is boosted to 20.73% with a *J*_SC_ of 23.71 mA cm^−2^, *V*_OC_ of 1.158 V, and FF of 75.5%. The steady-state photocurrent and stabilized power output are shown in Figure S5. The device achieved a champion stable output of more than 20.6%. The results of external quantum efficiency (EQE) and integrated photocurrent were also in good agreement with *J*_SC_ (Figure S6).

**Table 1:** The parameters of PSCs modified with different concentrations of TEMPO (scan speed: 100 mV/s).

**Device**	***V*_**OC**_ (V)**	***J*_**SC**_ (mA cm^**−2**^)**	**FF (%)**	**PCE (%)**	***V*_**max**_(V)**	**Rs (Ω cm^**2**^)**	**Rsh (Ω cm^**2**^)**
0 mg/ml	1.119	23.26	73.8	19.22	0.92	4.8	1065.75
1 mg/ml	1.151	23.61	74.5	20.24	0.95	3.22	1136.04
2 mg/ml	1.158	23.71	75.5	20.73	0.97	3.74	1171.3
4 mg/ml	1.126	23.28	74.2	19.47	0.93	4.61	948.32

The statistical photovoltaic parameters of the device and the average values are listed in Figure S7 and Table S3. The average *V*_OC_ increased from 1.10 ± 0.01 to 1.15 ± 0.01 V with the TEMPO concentration increased from 0 to 2 mg ml^−1^, while it decreased to 1.13 ± 0.01 V after further increasing the concentration to 4 mg ml^−1^. The increase of *V*_OC_ was due to the fact that TEMPO passivates the defects of perovskite films and suppresses the non-radiative recombination. The average *J*_SC_ values were 23.12 ± 0.27, 23.27 ± 0.36, 23.75 ± 0.26, and 23.22 ± 0.21 mA cm^−2^, for the devices without TEMPO and with 1, 2, and 4 mg ml^−1^ TEMPO, respectively. The average FF of devices without TEMPO increased from (0.73 ± 0.01) to (0.74 ± 0.01) after adding 2 mg mL^−1^ TEMPO. The average FF of devices with 1 mg mL^−1^ TEMPO and 2 mg mL^−1^ TEMPO was very similar to that of without TEMPO. The PCE of (18.70% ± 0.34%), (19.59% ± 0.36%), (20.26% ± 0.42%), and (19.05% ± 0.32%) for the PVSCs without TEMPO, with 1 mg mL^−1^, 2 mg mL^−1^, and 4 mg mL^−1^ TEMPO, respectively. In addition, the unencapsulated devices based on TEMPO exhibited excellent stability. We studied the trend of normalized PCE over time in ambient atmospheric under dark (relative humidity 50 ± 5%) (Figure 4C). TEMPO based devices retain 76% of their initial PCEs after aging 3,000 h showing a slow degradation in the air whereas the control devices only retain 42%. The average values of performance parameters of the aged device are listed in Table S5. The average PCE of TEMPO modulated devices after aging was (14.92% ± 0.29) compared with control devices (8.10% ± 0.16).

## Conclusions

In summary, we have successfully demonstrated a new kind of radical molecular 2,2,6,6-tetramethylpiperidine-1-oxyl for achieving efficient and stable PSCs. Lewis base of N-O radical group has a strong interaction with the Pb^2+^ ions which can form the radical Lewis base-acid adducts. These adducts can effectively passivate the defects at grain boundaries, thus suppressed non-radiative recombination. In addition, radical molecular can form the single electron hydrogen bond with FA^+^. As a result, under the optimized TEMPO doping amount of 2 mg/mL, we achieved a significant improvement of the PCE of n-i-p PSC device from 19.22 to 20.73%. Especially, a high *V*_OC_ of 1.158 V is achieved, which is 40 mV improve compared with control. This study provides a new class of molecules for perovskite additive engineering and lays a foundation for further exploring the application of free radicals in perovskite photovoltaic devices.

## Experimental

### Materials and Methods

Unless the special statement, all materials were purchased from Sigma-Aldrich or TCI and used without further purification. The SnO_2_ solution was purchased from Alfa Aesar (tin (IV) oxide, 15 wt% in H_2_O colloidal dispersion). Dimethylformamide (DMF, 99.8%), dimethyl sulfoxide (DMSO, 99.7%), ethyl acetate (EA, 99.8%) and Chlorobenzene (CB, 99.8%) were purchased from Acros; 4-tert-butyl pyridine (tBP, 96%), lithium bistrifluorosulfonyl imide (LiTFSI, 99.95%), lead iodide (PbI_2_, 99.99%) and lead bromine (PbBr_2_, 99%) were purchased from TCI. Methylammonium iodide (MAI), methylammonium chloride (MACl), methylammonium bromine (MABr) and 2,2′,7,7′-tetrakis-(N,N-di-p-methoxyphenylamine)-9,9′-spirobifluorene (spiro-MeOTAD, 99.5%) were purchased from Xi'an Polymer Light Technology Corp. Indium tin oxide (ITO, Transmission>95%) substrates were purchased from South China Science & Technology Company limited.

### Preparation of ETL Precursor Solutions

SnO_2_ nanoparticle precursor solutions were diluted by deionized water with a volume ratio of 1:5, stirred for 2 h, and filtered (0.22 μm, CA) before use.

### Preparation of Perovskite Precursor Solutions

The precursor solutions for (FAPbI_3_)_0.95_(MAPbBr_3_)_0.05_ perovskite (FA_0.95_MA_0.05_) (1.5 M) were prepared by dissolving 8.40 mg of MABr, 27.53 mg of PbBr_2_, 245.06 mg of FAI, 689.77 mg of PbI_2_, and 35.18 mg of MACl into a 1 mL mixture of DMF and DMSO (DMF:DMSO = 4:1, volume/volume). The perovskite precursor solutions were stirred for 2 h in the nitrogen-filled glove box. The perovskite precursor solutions were filtered (0.22 μm, PTFE) before use.

### Preparation of HTL Precursor Solutions

A Spiro-OMeTAD solution was prepared by dissolving Spiro-OMeTAD in chlorobenzene (72.3 mg), with the addition of 28.8 μl of 4-*tert*-butylpyridine (TBP), 17.5 μl of Li-bis(trifluoromethanesulfonyl)imide (Li-TFSI)/acetonitrile (520 mg/ml), stirred for 2 h and filtered (0.22 μm, PTFE) before use.

### Device Fabrication

Patterned indium tin oxide (ITO) glass substrates with a sheet resistance <15 Ω/sq were cleaned with detergent, deionized water, acetone, and ethanol sequentially. Each step was performed for 15 min. Before use, the ITO-coated glass was cleaned with plasma treatment for 5 min. Then the substrate was spin-coated with SnO_2_ ETL at 4,000 rpm for 30 s, followed by annealing air at 150°C for 30 min in ambient temperature. It is better to clean the substrate with plasma treatment for 5 min to improve the surface wetting. After that, the perovskite absorbers (FA_0.95_MA_0.05_) were deposited on the ETL substrates. The spin-coated process can be divided into two processes: with a spin rate of 1,000 rpm for 7 s for the first step and a spin rate of 5,000 rpm for 40 s for the second step. Thirty seconds after the second step, 140 μl of ethyl acetate was added to treat the perovskite films. Then, the perovskite films were annealed at 100°C for 30 min in a glove box which was filled with N_2_. After cooling down to room temperature, the HTL solution was coated on perovskite films by spin-coating at 3,000 rpm for 30 s with an accelerated speed of 2,000 rpm. Finally, the substrates coated with HTL were transferred into a vacuum chamber, and an 80 nm Au film was thermally evaporated as a counter electrode using a shadow mask with a base pressure of 4 × 10^−4^ Pa.

### Measurements and Characterization

A field-emission scanning electron microscope (SEM) (FEI Nova NanoSEM 450) was employed to measure the morphology patterns. The XRD spectra were measured by the X'pert PRO X-ray diffractometer using Cu Kα radiation under operating conditions of 40 kV and 40 mA from 10° to 50°. The thickness of the films was measured by a profilometer (DektakXT, Bruker). The UV-vis was measured by using an ultraviolet-visible near-infrared spectrophotometer (SolidSpec-3700). The IR spectra were recorded on a Bruker FTIR spectrometer. XPS measurements were carried out by an X-ray photoelectron spectroscope (Axis-Ultra DLD-600W). TRPL decay transients were taken at 810 nm from a HORIBA Scientific DeltaPro fluorometer using excitation with a 532 nm light pulse. The steady-state PL spectra were measured by a HORIBA Jobin Yvon LabRAM HR800s Raman spectrometer with a 532 nm wavelength excitation source. Besides, the device performance was characterized by a Keithley 2,400 source/meter and a Newport solar simulator (model 94043A) which offers the simulated AM 1.5G illumination of 100 mW cm^−2^; calibration was done using a NIST-certified monocrystalline Si solar cell (Newport 532 ISO1599). The *J*–*V* curves have been measured by reverse scans (1.2 to −0.1 V) with a scan rate of 100 mV s^−1^. A mask with a circular aperture (0.101 cm^2^) was used for *J*–*V* measurements.

Density functional theory (DFT) calculations were performed on the Gaussian 09 program by using the B3LYP and 6–31G^*^ basis set with vdW dispersion-corrected functionals (DFT-D3). The default spin mode was used to optimize the geometry of free radical molecules in the gas phase. Through the calculation of vibration frequency, the optimized molecular structure of free radical had no virtual frequency.

## Data Availability Statement

All datasets presented in this study are included in the article/supplementary material.

## Author Contributions

QP and XZ contributed equally to this work. The manuscript was written by QP, HZ, and XL. The idea of the study was conceived by HZ and XL, while QP and XZ conducted the device fabrication and characterization. XZ and LLi conducted the FTIR. SY and SZ conducted the XPS. YZ and LLuo conducted the UV-vis, PL, and TRPL measurements. All authors contributed to the article and approved the submitted version.

## Conflict of Interest

The authors declare that the research was conducted in the absence of any commercial or financial relationships that could be construed as a potential conflict of interest.
