# Supplementary material for: Radical Molecular Modulator for High-Performance Perovskite Solar Cells
Source: Front Chem. 2020 Sep 11;8:825. doi: 10.3389/fchem.2020.00825 (PMC7516026; doi:10.3389/fchem.2020.00825)
Supplement: Supplementary file 1 [file Table_1.DOCX]

***Supplementary Material***


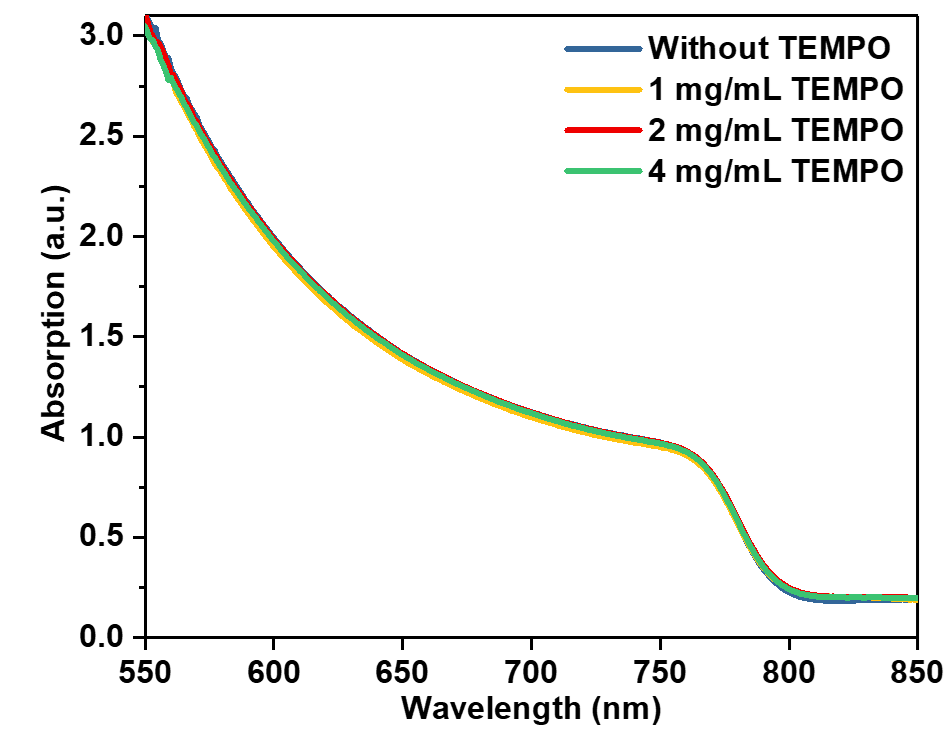


**Fig. S1**. UV–vis absorbance of perovskite films with different concentration ratio of TEMPO.


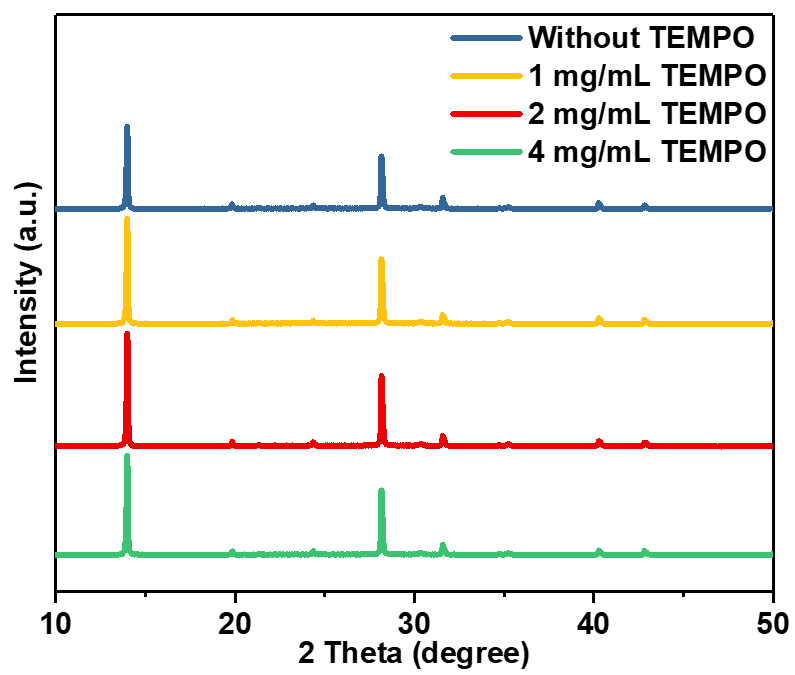


**Fig. S2**. XRD patterns of perovskite films with different concentration ratio of TEMPO.


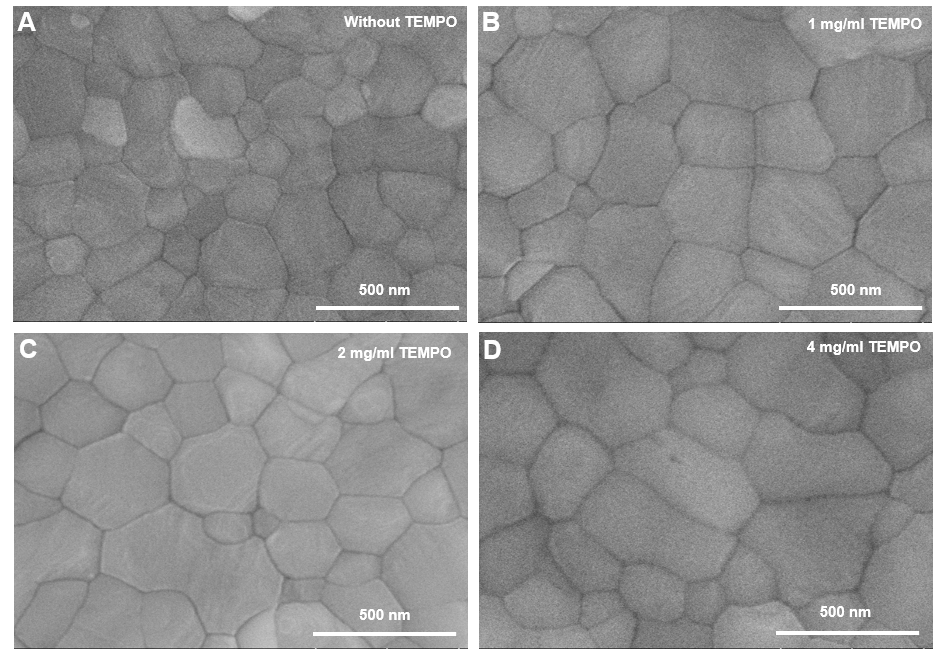


**Fig. S3**. Plane-view SEM images of perovskite films with different concentration ratio of TEMPO.

**Fig. S4**. Molecular structure of radical small molecules with different functional groups.


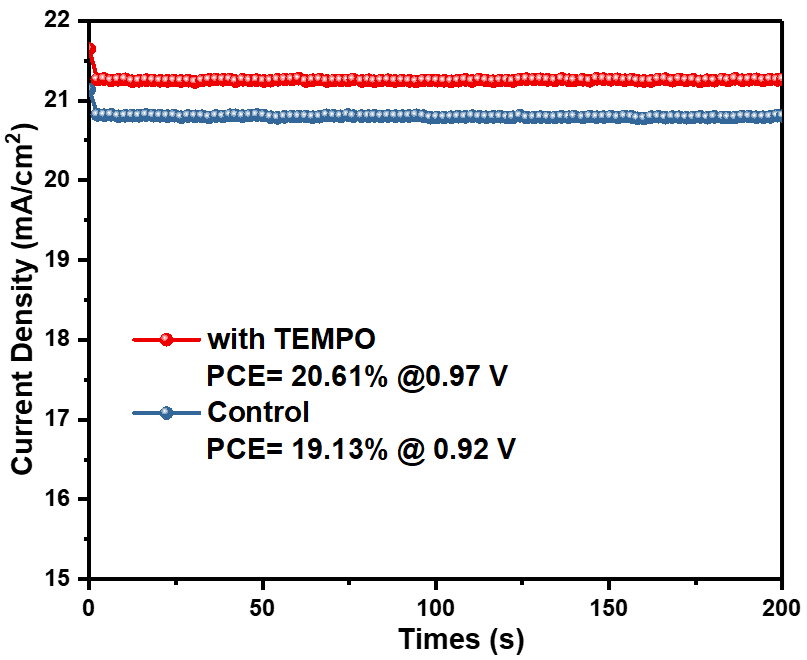


**Figure S5.** Steady-state photocurrent and stabilized power output of the champion device with and without TEMPO.


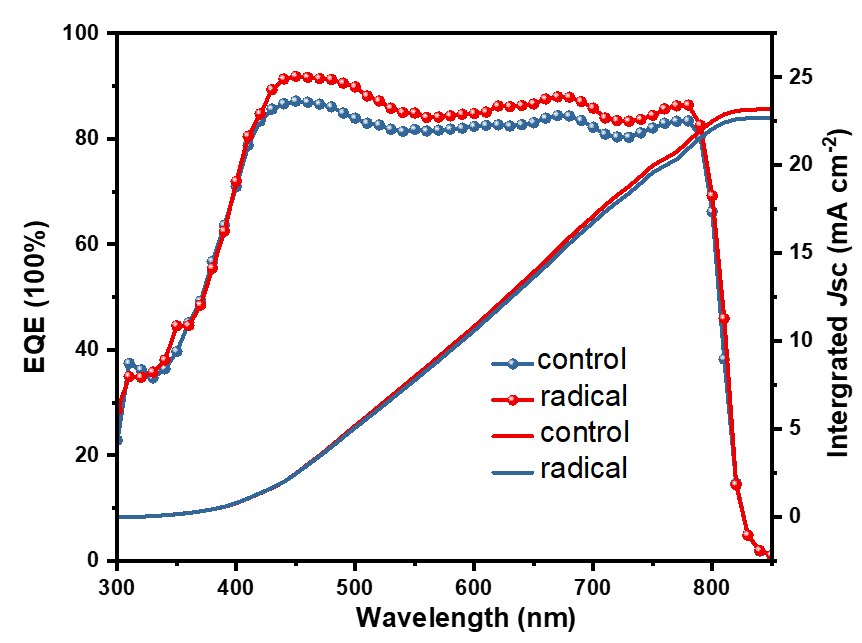


**Figure S6.** EQE and integrated current density of the device with and without TEMPO.


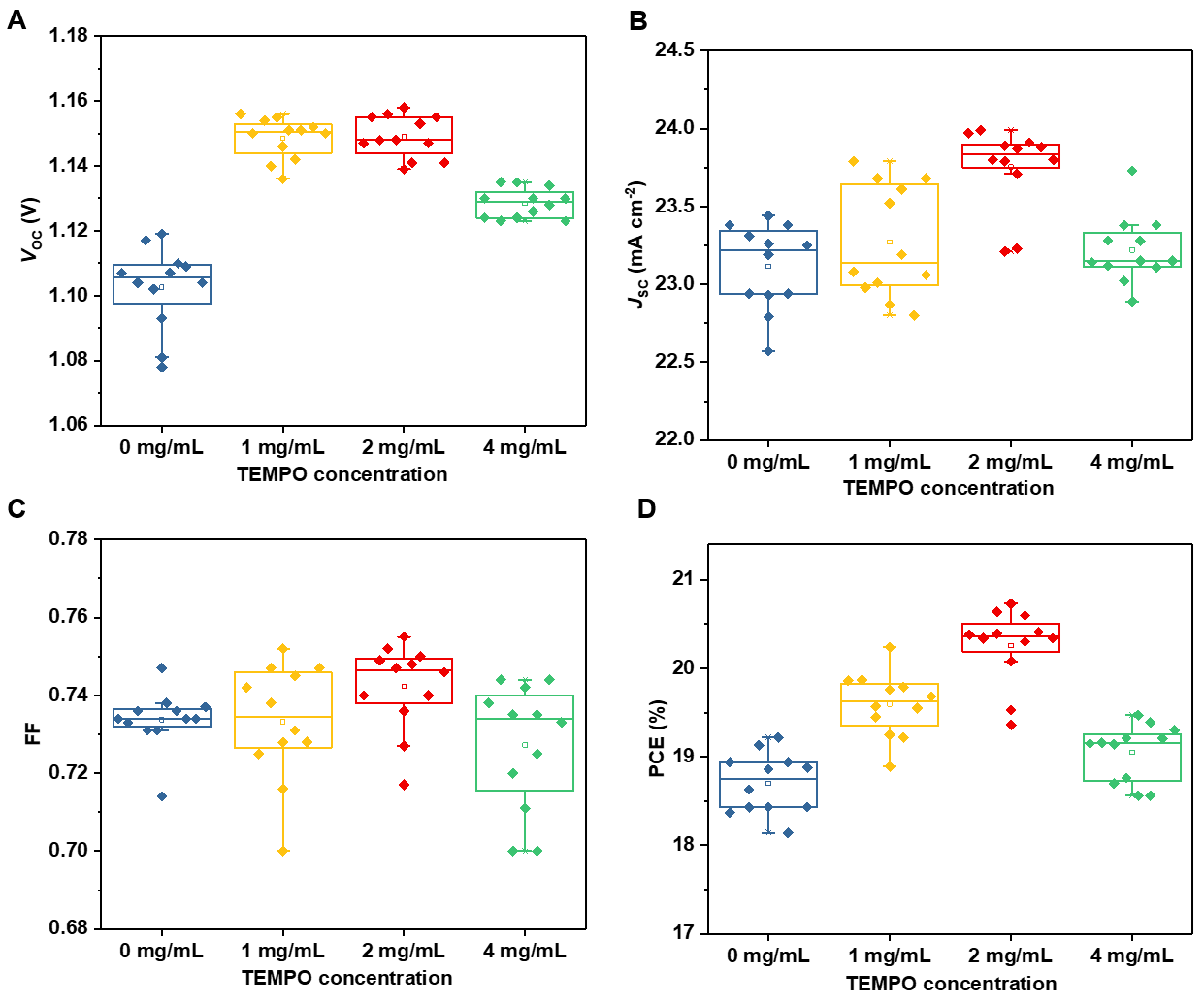


**Figure S7.** Statistic a) J_SC_, b) V_OC_, c) FF, and d) PCE of devices with different concentration of TEMPO. The statistical data were obtained from 12 cells for each ratio.

**Table S1** The fitting results of TRPL spectra of the perovskite films modified without and with TEMPO

| Entry | τ_1_ (ns) | τ_2_ (ns) | A_1_*^a^* (%) | A_2_*^a^* (%) | τ*^b^* (ns) |
| --- | --- | --- | --- | --- | --- |
| Without TEMPO | 70.87 | 387.31 | 4.27 | 95.73 | 384.75 |
| 1 mg/mL TEMPO | 146.16 | 539.08 | 4.02 | 95.98 | 534.67 |
| 2 mg/mL TEMPO | 159.57 | 705.18 | 1.97 | 98.03 | 702.71 |
| 4 mg/mL TEMPO | 82.19 | 454.13 | 4.25 | 95.75 | 451.17 |

*^a^*A_1_ and A_2_ are the fractions of two PL decay components respectively; *^b^*τ is average PL lifetime.

**Table S2** The parameters of control device and PSCs modified with different radical small molecules

| Device | *V*_OC_ (V) | *J*_SC_ (mA cm^−2^) | FF (%) | PCE (%) |
| --- | --- | --- | --- | --- |
| Control | 1.073 | 21.45 | 70.3 | 16.18 |
| TEMPO | 1.084 | 21.87 | 73.1 | 17.33 |
| TEMPO-NH_2_ | 0.984 | 21.12 | 48.6 | 10.09 |
| TEMPO-OH | 1.056 | 21.26 | 59.4 | 13.33 |

**Table S3** The average parameters of PSCs modified without TEMPO, with 1 mg mL^–1^, 2 mg mL^–1^ and 4 mg mL^–1^ TEMPO

| Device | *V*_OC_ (V) | *J*_SC_ (mA cm^−2^) | FF (%) | PCE (%) |
| --- | --- | --- | --- | --- |
| 0 mg/mL | 1.10±0.01 | 23.12±0.27 | 0.73±0.01 | 18.70±0.34 |
| 1 mg/mL | 1.15±0.01 | 23.27±0.36 | 0.73±0.02 | 19.59±0.36 |
| 2 mg/mL | 1.15±0.01 | 23.75±0.26 | 0.74±0.01 | 20.26±0.42 |
| 4 mg/mL | 1.13±0.01 | 23.22±0.21 | 0.73±0.02 | 19.05±0.32 |

**Table S4.** The initial and final photovoltaic parameters of a batch of unsealed perovskite devices modified with TEMPO measured under simulated AM 1.5G solar light at 100 mW cm^−2^ intensity during the stability test under low humidity and dark conditions.

| Cell No | *V*_OC_ (V) | *J*_SC_ (mA cm^−2^) | FF | PCE (%) |
| --- | --- | --- | --- | --- |
| 1*^I^* | 1.16 | 23.23 | 0.75 | 20.21 |
| 2*^I^* | 1.14 | 23.87 | 0.72 | 19.59 |
| 3*^I^* | 1.15 | 23.89 | 0.75 | 20.61 |
| 4*^I^* | 1.15 | 23.21 | 0.73 | 19.48 |
| 5*^I^* | 1.15 | 23.79 | 0.75 | 20.52 |
| Average*^I^* | 1.15 ± 0.01 | 23.60 ± 0.35 | 0.74 ± 0.01 | 20.08 ± 0.52 |
| 1*^F^* | 1.06 | 18.62 | 0.76 | 15 |
| 2*^F^* | 1.07 | 17.76 | 0.78 | 14.82 |
| 3*^F^* | 1.11 | 17.16 | 0.8 | 15.24 |
| 4*^F^* | 1.01 | 18.62 | 0.77 | 14.48 |
| 5*^F^* | 1.08 | 18.34 | 0.76 | 15.05 |
| Average*^F^* | 1.07 ± 0.04 | 18.10 ± 0.63 | 0.77 ± 0.02 | 14.92 ± 0.29 |

*^I^* The initial photovoltaic parameters during the stability test over 3000 h.

*^F^* The final photovoltaic parameters during the stability test over 3000 h.

**Table S5.** The initial and final photovoltaic parameters of a batch of unsealed perovskite devices modified without TEMPO measured under simulated AM 1.5G solar light at 100 mW cm^−2^ intensity during the stability test under low humidity and dark conditions.

| Cell No | *V*_OC_ (V) | *J*_SC_ (mA cm^−2^) | FF | PCE (%) |
| --- | --- | --- | --- | --- |
| 1*^I^* | 1.08 | 23.19 | 0.74 | 18.53 |
| 2*^I^* | 1.11 | 23.25 | 0.73 | 18.84 |
| 3*^I^* | 1.08 | 22.79 | 0.74 | 18.21 |
| 4*^I^* | 1.10 | 23.38 | 0.73 | 18.77 |
| 5*^I^* | 1.09 | 22.94 | 0.73 | 18.25 |
| Average*^I^* | 1.09 ± 0.01 | 23.11 ± 0.24 | 0.73 ± 0.01 | 18.52 ± 0.29 |
| 1*^F^* | 0.96 | 12.89 | 0.65 | 8.04 |
| 2*^F^* | 0.96 | 12.51 | 0.69 | 8.29 |
| 3*^F^* | 1.01 | 12.28 | 0.64 | 7.94 |
| 4*^F^* | 0.92 | 13.19 | 0.68 | 8.25 |
| 5*^F^* | 0.93 | 12.59 | 0.68 | 7.96 |
| Average*^F^* | 0.96 ± 0.04 | 12.69 ± 0.35 | 0.67 ± 0.02 | 8.10 ± 0.16 |

*^I^* The initial photovoltaic parameters during the stability test over 3000 h.

*^F^* The final photovoltaic parameters during the stability test over 3000 h.
